# Genomics of Adaptation during Experimental Evolution of the Opportunistic Pathogen *Pseudomonas aeruginosa*

**DOI:** 10.1371/journal.pgen.1002928

**Published:** 2012-09-13

**Authors:** Alex Wong, Nicolas Rodrigue, Rees Kassen

**Affiliations:** 1 Department of Biology, Carleton University, Ottawa, Canada; 2 Department of Biology, University of Ottawa, Ottawa, Canada; 3 Eastern Cereal and Oilseed Research Centre, Agriculture and Agri-Food Canada, Ottawa, Canada; University of Toronto, Canada

## Abstract

Adaptation is likely to be an important determinant of the success of many pathogens, for example when colonizing a new host species, when challenged by antibiotic treatment, or in governing the establishment and progress of long-term chronic infection. Yet, the genomic basis of adaptation is poorly understood in general, and for pathogens in particular. We investigated the genetics of adaptation to cystic fibrosis-like culture conditions in the presence and absence of fluoroquinolone antibiotics using the opportunistic pathogen *Pseudomonas aeruginosa*. Whole-genome sequencing of experimentally evolved isolates revealed parallel evolution at a handful of known antibiotic resistance genes. While the level of antibiotic resistance was largely determined by these known resistance genes, the costs of resistance were instead attributable to a number of mutations that were specific to individual experimental isolates. Notably, stereotypical quinolone resistance mutations in DNA gyrase often co-occurred with other mutations that, together, conferred high levels of resistance but no consistent cost of resistance. This result may explain why these mutations are so prevalent in clinical quinolone-resistant isolates. In addition, genes involved in cyclic-di-GMP signalling were repeatedly mutated in populations evolved in viscous culture media, suggesting a shared mechanism of adaptation to this CF–like growth environment. Experimental evolutionary approaches to understanding pathogen adaptation should provide an important complement to studies of the evolution of clinical isolates.

## Author Summary

Pathogens face a hostile and often novel environment when infecting a new host, and adaptation to this environment can be critical to a pathogen's survival. The genetic basis of pathogen adaptation is in turn important for treatment, since the consistency with which therapies succeed may depend on the extent to which a pathogen adapts via the same routes in different patients. In this study, we investigate adaptation of the bacterium *Pseudomonas aeruginosa* to laboratory conditions that resemble the lungs of cystic fibrosis patients and to quinolone antibiotics. We find that a handful of genes and genetic pathways are repeatedly involved in adaptation to each condition. Nonetheless, other, less common mutations can play important roles in determining fitness, complicating strategies aimed at reducing the prevalence of antibiotic resistance.

## Introduction

In the mid-1800's, Louis Pasteur advised microbiologists to think of the human body as a “culture vessel” for microbes, in the context of understanding immunity [1]. Pasteur's approach has been revised and updated several times [2], [3], with a recent review encouraging researchers to be attentive to the effects of different *in vivo* carbon sources on bacterial metabolism and physiology [2]. Pasteur's advice is particularly relevant for an understanding of the evolution of disease-causing microbes. Natural selection may be imposed by the particular nutritional and metabolic resources available in a given tissue, the innate and adaptive immune systems, and, in the past 80 or so years, by antibiotics or anti-virals. Many pathogens – particularly opportunistic pathogens, emerging pathogens, and microbes causing chronic disease – are faced with a novel and hostile growth environment to which they must adapt or face extinction. Colonization and establishment of an infection in a new host or host species can thus be interpreted as a specific instance of a more general process of adaptation to a novel environment.

Understanding adaptive processes in pathogen populations, and in particular characterizing the variety of genetic routes to adaptation, is important for developing effective treatment strategies. Take as an example the management of antibiotic resistance. Resistance is often thought to be costly, in the sense that resistant strains should be less fit than susceptible strains in the absence of antibiotic. If so, then attempts to reduce the frequency of resistance in patient populations by stopping the use of an antibiotic should afford sensitive strains an advantage, and so prolong the utility of an antibiotic for treatment. Antibiotic cessation has met with mixed success (e.g., [4]–[6]), however, either because some resistance mutations actually pay little or no cost, or because second site mutations that restore fitness without compromising resistance are common. The management of antibiotic resistance in patient populations depends crucially on which of these two mechanisms is more often responsible for the persistence of resistance.

The last 15 years have seen a number of studies of *in vivo* genome evolution in select pathogens, primarily viruses (e.g., [7], [8]) and bacteria (e.g., [9], [10]), that shed vital insight onto the genetic changes that occur during epidemics or chronic infections. The importance of these changes for pathogen fitness in a host can be difficult to ascertain, however, because it is rarely possible to establish with certainty that the observed mutations are adaptive, since some neutral or deleterious mutations may accumulate through drift or by hitchhiking with adaptive mutations. Moreover, it can be difficult to obtain sufficient *in vivo* samples to ask questions about the repeatability of *in vivo* evolution – that is, how often pathogens take the same adaptive routes in independent patients or populations.

For these reasons we have turned to a complementary approach, laboratory selection experiments, to provide an understanding of the broad patterns and principles of pathogen evolution. In a typical microbial experimental evolution protocol, many populations are founded from a single genotype, and are propagated serially or in a chemostat for tens, hundreds, or thousands of generations (reviewed in [11]). By maintaining multiple replicate populations in each of two or more environments (e.g., antibiotic treated vs. not antibiotic treated), the effects of a treatment can be systematically investigated in a manner that is often inaccessible with *in vivo* samples. Experimental evolution has by now a rich history in studying basic evolutionary processes (e.g., [11]–[14] for reviews), as well as more applied topics such as the evolution of antibiotic resistance [15], [16] and of virulence [7]. In addition, experimental evolution has significant potential as an investigative tool for elucidating basic biological processes [17], [18]. With the development of technologies that allow the rapid and affordable sequencing of entire bacterial genomes, an increasing number of studies have sought to describe the genomic basis of laboratory adaptation (reviewed in [19]).

Here we use a combination of experimental evolution and whole-genome sequencing (WGS) to investigate the initial stages of pathogen adaptation using the bacterium *Pseudomonas aeruginosa*. This gram-negative bacterium is widely distributed in nature [20], and is an important opportunistic pathogen. *P. aeruginosa* can cause acute infections of wounds, burns and of lungs, and is frequently implicated in nosocomial infections. Moreover, *P. aeruginosa* is an important pathogen of individuals with cystic fibrosis (CF), with approximately 60–70% of Canadian adults with CF harbouring this bacterium [21]. *P. aeruginosa* chronically infects the CF lung, and once the infection is established, it is virtually impossible to eradicate: Intensive antibiotic regimens are effective at reducing symptoms, but almost never succeed in clearing the infection entirely.

*P. aeruginosa* populations that have persisted for long periods of time in the lungs of individuals with CF show characteristic signatures of adaptation to this novel culture environment. Recent studies have documented patterns of parallel evolution at the level of phenotype, gene expression, and genotype [10], [22]–[25], indicating repeatable patterns of long-term adaptation to the CF lung. For example, CF lung sputum is highly viscous, and *P. aeruginosa* typically grows as an unattached biofilm, or microcolony, in this environment [26]. While environmental isolates of *P. aeruginosa* are motile, long-term CF colonists show evidence of adaptation to the sessile lifestyle of the microcolony, including reduced motility, and a morphological shift to small colony variants (SCVs) on agar plates [27], [28]. Increased intracellular levels of cyclic di-GMP are thought to be important for this adaptive shift [27]–[29], but the causative mutations have yet to be fully elucidated. Other characteristic changes include mutations associated with reduced virulence, presumably to avoid detection by the host immune system, and increased small molecule efflux that can afford resistance to antibiotics commonly used with CF patients [10].

Given evidence of long-term adaptation during chronic infection in *P. aeruginosa*, we have examined the genomic basis of adaptation to CF-like culture conditions and to fluoroquinolone antibiotics through WGS of experimentally evolved *P. aeruginosa* isolates. Our primary aim is to describe the genetic changes underlying adaptation to this novel environment, and to ask how repeatable these changes are. In addition, we also investigate the genetic architecture of the costs of resistance: When antibiotic resistance evolves, how often is it costly, and what mutations underlie those costs? Our data allow us to quantify the nature and extent of parallel genomic evolution and, in so doing, provide a unique view of the variety of genetic routes taken during adaptation to a medically relevant novel environment.

## Results/Discussion

### Adaptation to culture environments and to ciprofloxacin

In our selection experiment, we manipulated the bacterial growth environment so as to resemble the CF lung with respect to nutrition, viscosity, and antibiotic treatment. Populations of *P. aeruginosa* were evolved in synthetic cystic fibrosis sputum (scfm; [30]) for 8 days in the presence or absence of ciprofloxacin (Cip) and/or mucin. Scfm is a defined medium resembling the nutritional environment of the CF lung [30]. Ciprofloxacin was added at a concentration comparable to that found in the sputum of CF patients (1 ug/ml; [31]). Mucin increases the viscosity of the culture medium, and is meant to mimic the high viscosity of CF sputum [32], [33]. *In vivo*, viscous sputum is thought to support the growth of *P. aeruginosa* in unattached biofilms, called microcolonies [26], [34], and similar structures have been observed in mucin-supplemented media (e.g., [32], [33]). Mucin was added at 10 g/L. Mucin may also act as a source of nutrients. The selection experiment comprised a fully factorial design giving four selection environments: scfm alone, scfm+Cip, scfm+mucin, and scfm+mucin+Cip; 12 replicate populations were propagated in each environment. Populations were maintained in a 37°C shaking incubator in 1.5 ml of medium, with serial transfer at a 1∶61 dilution every 24 hours, with approximately 5.9 generations of growth per day (47.5 generations in total).

Since the CF lung – and by extension laboratory media designed to mimic aspects of the CF lung – is a unique growth environment for bacteria, our evolved *P. aeruginosa* populations are expected to adapt to this novel habitat. Adaptation is also expected to occur in response to ciprofloxacin through the selection of mutations conferring resistance. Our experimental design allows us to disentangle these two effects, with fitness in the absence of antibiotic serving as a measure of adaptation to the growth medium, and changes in resistance to ciprofloxacin indicating adaptation to the presence of this antibiotic. Since populations may harbour extensive genetic and phenotypic variation, we measured resistance and fitness for evolved populations, as well as for a single genotype isolated from each population.

**Figure 1:**
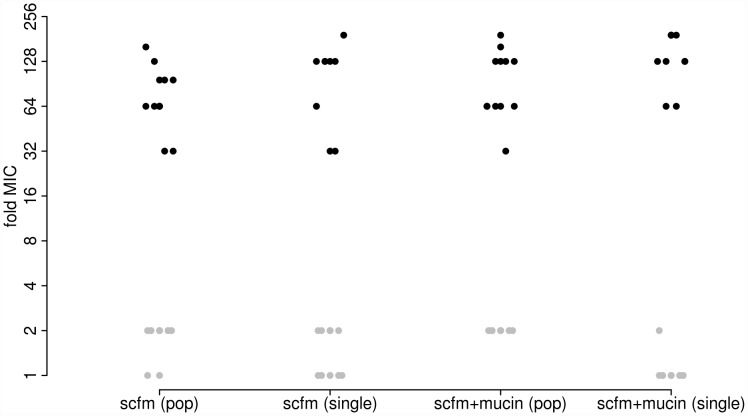
Minimum inhibitory concentration (MIC) to ciprofloxacin for each of 48 experimentally evolved populations (“pop”) or single gentoypes (“single”). Populations were evolved in the presence (black) or absence (grey) of 1 µg/ml ciprofloxacin for 8 days (∼50 generations). Two different media were used, as indicated on the X-axis.

As expected, antibiotic resistance evolved in the presence of ciprofloxacin at both the population and genotype levels (Figure 1). Populations evolved in the presence of Cip showed a 32-fold to 192-fold increase in minimal inhibitory concentration (MIC) over the ancestral genotype Pa14, whereas those evolved in the absence of Cip increased MIC by no more than 2-fold. Single genotypes isolated from each population gave similar results: genotypes evolved in Cip had MICs ranging from 32-fold to 192-fold greater than the ancestor.

To detect adaptation to the growth medium we assayed the fitness of evolved populations and genotypes in the absence of antibiotic using direct, head-to-head competitions against Pa14 (see Materials and Methods). We interpret the population-level assays as a measure of the extent of adaptation achieved, since these reflect the average increase in fitness of all genotypes present at the end of the experiment. The single-genotype assays provide a measure of adaptation for the same genotypes we have sequenced (see below). Note that there will be a close correspondence between measures of fitness at the population and single-genotype levels only if the population is genetically uniform, as expected under a model of periodic strong selection. If, however, the population is genetically polymorphic, perhaps because mutation supply rates (the product of population size, *N*, and mutation rate, *u*) are high or distinct genotypes are maintained by negative frequency dependent selection, then adaptation detected at the level of the population may not be accurately predicted by assays of fitness from single genotypes.

**Figure 2:**
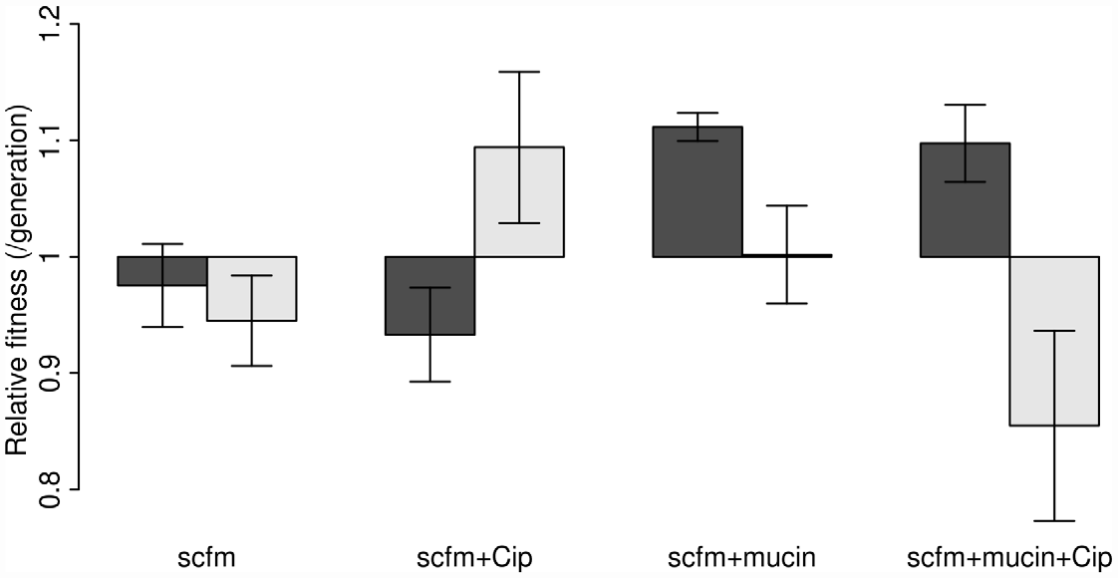
Competitive fitness of experimentally evolved populations (dark bars) or single genotypes (light bars). Fitness was measured in the absence of antibiotic via direct competitions with a lacZ marked ancestral strain (Pa14). The height of each bar indicates mean fitness for 12 evolved populations (genotypes), with the error bar giving +/−1 SE. Competitions were carried out in scfm for populations (genotypes) evolved in scfm or in scfm+ciprofloxacin, and in scfm+mucin for populations (genotypes) evolved in scfm+mucin or in scfm+mucin+ciprofloxacin. Fitness below one indicates low fitness relative to the ancestor, while fitness above one indicates an overall benefit in the absence of antibiotic.

Our results are shown in Figure 2, where the dark bars represent the extent of adaptation by entire populations and the light bars adaptation by single genotypes. Evolved populations adapted to the growth medium without antibiotic only when mucin was present in the medium. In the absence of mucin, there was either no response to selection (scfm) or a significant cost to adaptation to Cip (scfm+Cip; ANOVA: *P* = 2.9×10−5; Table 1). Thus, the presence of mucin in the environment affords a greater opportunity for rapid adaptation.

**Table 1:** Effects of selection environment on fitness in the absence of antibiotic.

	Population		Single genotypes	
Factor	F-value	*P*-value	F-value	*P*-value
Medium	21.76	**2.89×10^−5^**	2.03	0.16
Antibiotic	0.76	0.39	0.01	0.93
Medium*antibiotic	0.19	0.66	6.72	**0.013**

The single genotype fitness data are more mixed and do not correspond well with the population-level fitness assays (Figure S1), suggesting the presence of substantial amounts of genetic diversity within populations. We saw no consistent effect of mucin or of antibiotic on adaptation to the growth medium, as indicated by a lack of main effect for either of these factors by ANOVA (Table 1). There was, however, a significant interaction between medium and antibiotic (ANOVA: *P* = 0.013; Table 1), reflecting the observation that scfm+Cip-evolved genotypes were on average more fit than the ancestor (mean relative fitness *w* = 1.09/generation), whereas the scfm+mucin+Cip-evolved genotypes were on average less fit than the ancestor (mean *w* = 0.86/generation). This interpretation is reinforced by a lack of correlation between genotypes and populations for MIC, for which there was little correspondence between the level of resistance (Figure S2).

Taken together, these results suggest two important conclusions about short-term adaptation to a CF lung-like environment: (1) adaptation does occur, and it is driven primarily by the presence of mucin; and (2) substantial genetic diversity is likely to be present in evolving populations shortly after colonization, a result consistent with the observation that *P. aeruginosa* isolates from CF patients can often be highly diverse [10], [35], [36].

### Whole-genome sequencing of evolved genotypes

In order to gain insight into the genetic causes of adaptation, we sequenced the genomes of the pure genotypes assayed above, with one genotype sampled from each of the 48 evolved populations (that is, a single genotype from each population evolved in scfm alone, scfm+ciprofloxacin, scfm+mucin, and scfm+mucin+ciprofloxacin), as well as of our laboratory's isolate of the ancestral strain Pa14. We obtained a median coverage of ∼56-fold per genotype (mean = 55.5; range 31.8–85.4) on the Illumina platform, using 75-bp paired-end reads. Given that a previous study suggested that 15–20-fold coverage is sufficient for identifying a modest number of mutations in laboratory selected microbial strains [37], the depth of coverage we achieved should allow us to identify all SNPs and small indels throughout most of the genome. In addition, the sequenced genomes were surveyed for large insertion/deletion events, such as mobile element insertions or excisions. We were unable to survey ∼0.53% of the genome in each strain due to low coverage (defined as less than five reads covering a given nucleotide).

Across all 48 evolved strains, we identified 98 SNPs and small indels (mean 2.04/strain) not present in the ancestor (Table S1 lists all mutations and their predicted functional consequences). These mutations represented 77 unique changes, affecting a total of 44 genes and 4 putatively intergenic regions. No large insertion/deletion events were found using BRESEQ [38]. Two genotypes, both isolated from the scfm+mucin+Cip treatment, bore lesions in *mutS* and were thus likely mutator strains, an inference supported by the relatively high number of mutations found in these strains (one carried 30 mutations, and the other carried 4, representing the 1st and 3rd ranked genotypes in terms of number of mutations), as well as by an extreme transition∶transversion bias amongst point mutations (all 26 point mutations found in these two strains were transitions), which is characteristic of *mutS* mutants [39]. If these putative mutator strains are omitted, we found 64 mutations (44 unique changes) affecting 20 genes and 1 intergenic region (Figure S3). These mutations included 41 point mutations and 23 insertion/deletions (indels).

**Figure 3:**
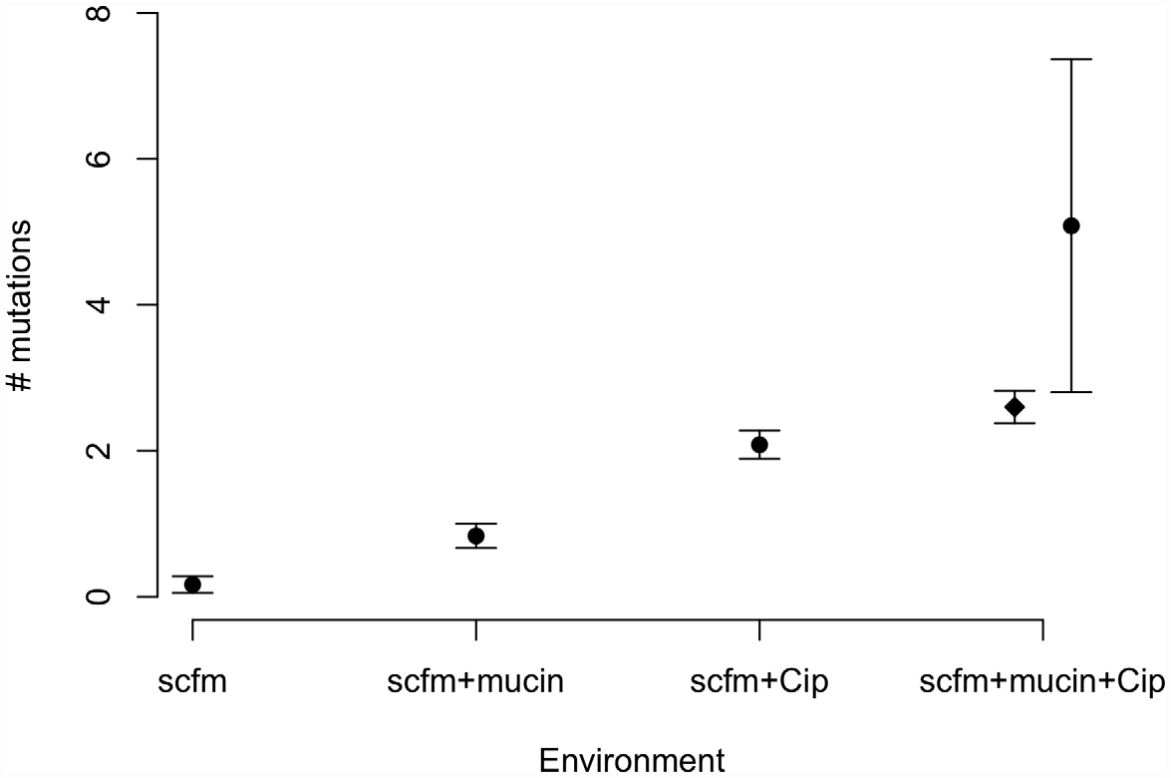
Numbers of mutations identified in evolved genotypes. Mean number of mutations by treatment, with error bars giving +/−1 SE. In the “scfm+mucin+Cip” treatment, the filled circle represents all evolved genotypes, and the filled diamond represents the ten non-mutator genotypes.

Genotypes evolved in the presence of ciprofloxacin or mucin carried more mutations on average than genotypes not evolved with antibiotic (Figure 3). Interestingly, genotypes from the most complex environment, containing both ciprofloxacin and mucin, carried more mutations than any other environment, on average. This result is broadly consistent with the idea that the number of mutations involved in adaptation increases with the number of distinct niche dimensions in the environment, an interpretation supported by both antibiotic and presence/absence of mucin being significant predictors of the number of mutations identified (ANOVA, mutators excluded; medium: *F* = 8.6, *P* = 0.005; antibiotic: *F* = 111.8, *P* = 2×10^−13^).

Previous studies of the genomic basis of adaptation in experimentally evolved bacterial populations have detected, on average, 1.07 mutations/100 generations (range: 0.09–3.94; [40]). The numbers of mutations observed after ∼48 generations in our antibiotic-evolved genotypes (mean 2.1 and 2.6 in scfm and scfm+mucin, respectively) are thus substantially higher than observed in previous studies. This difference probably reflects the strong selection imposed by antibiotic treatment, as opposed to the weaker selection commonly observed in resource-adaptation experiments, combined with sufficiently large population sizes to ensure the availability of multiple beneficial mutations in the same population or even the same genome [41]. Notably, the rate of accumulation of adaptive mutations observed here is consistent with theoretical models of substitution under strong selection that show expected fixation times of 50 generations or less for mutations with large selection coefficients (see Figure S4 from [42]). At the opposite end of the spectrum, very few mutations were detected in our scfm populations, with 10 genotypes bearing no mutations, and 2 genotypes carrying a single mutation each. This result is consistent with the lack of fitness response observed above (Figure 2) and is broadly consistent with the theoretical expectation under neutrality, whereby the expected fraction of 6.5 Mb genomes with zero mutations after 48 generations should be 0.73–0.97, depending on the per base pair mutation rate (taken as 1×10^−10^ to 1×10^−9^ for these estimates; [43]).

Broad patterns of nucleotide variability suggest that natural selection has played an important role in shaping the observed spectrum of mutations. Amongst the 41 point mutations observed in the non-mutator strains, 39 were nonsynonymous, 1 was synonymous, and 1 was putatively non-coding. Since approximately 1/3 of random coding changes are expected to be synonymous, the lack of synonymous mutations is consistent with natural selection favouring a substantial fraction of the observed mutations in the non-mutators. Using a randomization approach (see Materials and Methods), we find that both the excess of non-synonymous mutations, and the paucity of synonymous mutations, are highly significant (Figure S4; *P*<0.0005). By contrast, many more synonymous mutations were observed in the putative mutator strains, with 15 non-synonymous, 8 synonymous, 8 genic frame-shifts, and 3 intergenic mutations identified in the 2 putative mutators. The observed counts of non-synonymous and synonymous mutations in these mutators are not significantly different than expected by chance (non-synonymous: *P* = 0.30; synonymous: *P* = 0.43), suggesting that many more mutations are neutral and that these strains show a general and unbiased increase in mutation rate. The observed number of intergenic mutations (3) in the mutator strains is significantly higher than expected by chance, however (*P* = 0.011), suggesting that at least one of these mutations has been driven by selection.

### Genetic basis of adaptation to ciprofloxacin

**Figure 4:**
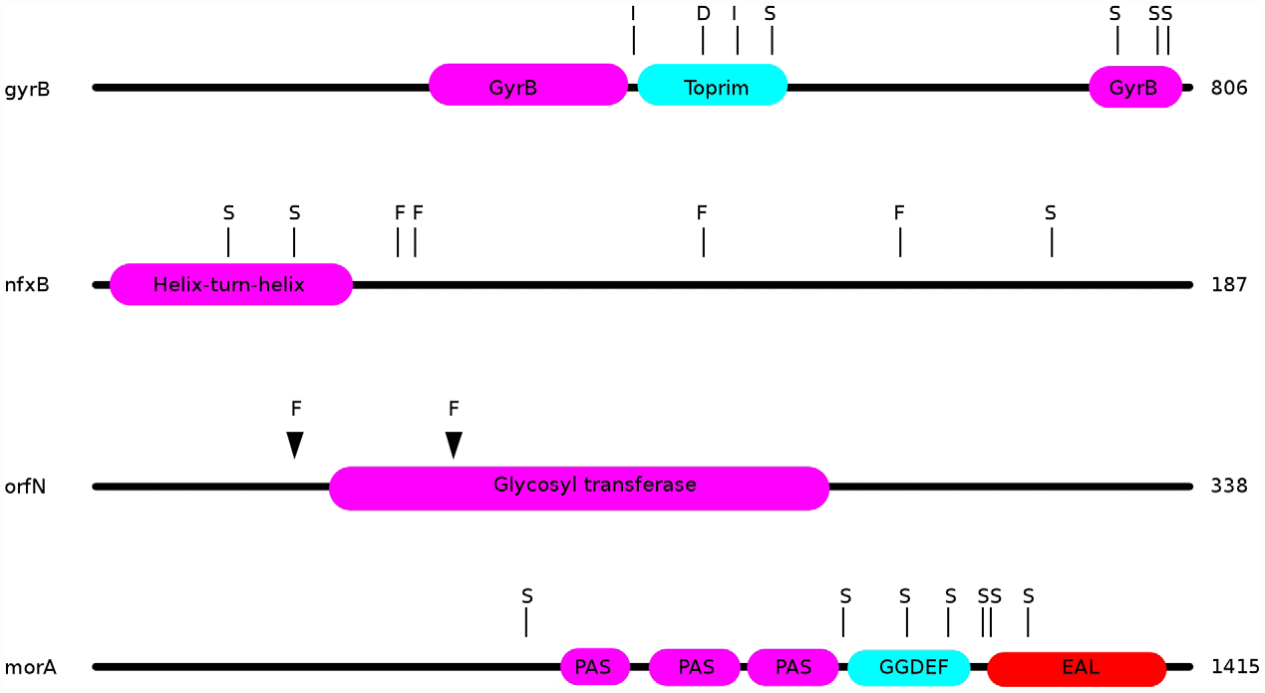
Locations of mutations in the ciprofloxacin resistance proteins nfxB, orfN, gyrB, and in the putatively mucin-adaptive protein morA. “S”: location of a single nucleotide polymorphism; “F”: frameshift; “I”: in-frame insertion; “D”: in-frame deletion.

Observed changes in ciprofloxacin MIC and in fitness are attributable to some or all of the mutations identified by WGS. For example, in the ciprofloxacin-evolved strains, we observed multiple mutations in the known fluoroquinolone-resistance genes *gyrA*, *gyrB*, and *nfxB*. Amongst 24 genotypes from populations evolved in the presence of ciprofloxacin, 20 bore mutations in *nfxB*, 9 carried mutations in *gyrB*, and 4 genotypes bore *gyrA* mutations. Each of the *gyrA* mutations is a known resistance mutation affecting its quinolone-resistance determining region (QRDR; [44], [45]), with one strain carrying a T83I mutation, two with D87G, and one with a D87N mutation. The *gyrB* mutations were dispersed throughout this gene, with 6 different lesions amongst the 9 strains (Figure 4). In *nfxB*, loss of function mutations would be expected to be prevalent, since inactivation of this transcriptional repressor results in up-regulation of the MexCD-OprJ efflux pump (e.g., [46]). Concordant with this expectation, 8 distinct mutations were found in *nfxB* among the 20 genotypes bearing mutations (Figure 4). Interestingly, three sites were mutated in multiple strains (T39P in 3 strains, in a predicted helix-turn-helix DNA-binding domain; E146K in 5 strains; G180S in 8 strains), providing further evidence that these mutations are adaptive.

Additionally, 7 ciprofloxacin-resistant genotypes carried mutations in the gene *orfN*, 6 being isolated from populations evolved in scfm+Cip. *orfN* encodes a predicted glycosyl transferase, and is necessary for the glycosylation of type A flagellins [47]. 6 of the *orfN* mutants carried a single base pair deletion in a poly-G repeat, leading to the introduction of a premature stop codon. The predicted mutant protein is truncated after 53 amino acid residues (vs 338 for the wild-type protein). The seventh *orfN* mutant carries a single base-pair deletion in a poly-T repeat, leading to a truncated protein of 133 residues. The predicted mutant proteins are truncated before or in the glycosyl transferase domain, suggesting that the *orfN* mutations are likely to be loss-of-function mutations (Figure 4). While this gene has not previously been associated with fluoroquinolone resistance, this observation of extensive parallel evolution strongly suggests that *orfN* mutants have increased fitness in the presence of ciprofloxacin.

To obtain further evidence for an effect of *orfN* and other putative novel resistance mutations on Cip resistance, we surveyed isolates from evolving populations from early time points and assayed their MICs in the genetic backgrounds in which they arose. This approach allows us to sample candidate genes relatively quickly in the context in which they evolved. For *orfN* mutants, we sampled single colony isolates from early time points (days 3–5 of the evolution experiment) from populations where an *orfN* mutation was observed at day 8. Early time-point isolates were sequenced at all genes bearing a SNP at day 8, and clones bearing only an *orfN* mutation were selected. In this way, we identified several apparent single *orfN* mutants: 2 from population scfm-A5 at day 3, and 1 from population scfm-D6 at day 5. As expected each of these putative single mutants showed a 32-fold elevation in ciprofloxacin MIC in comparison to the ancestral Pa14 genotype, suggesting that *orfN* is a novel resistance gene.

While the observation of parallel evolution at *nfxB*, *gyrA*, *gyrB*, and *orfN* is indicative of natural selection acting on these genes, 12 of the mutations identified in the non-mutator strains appeared in only a single isolate each (Figure S5). Such mutations may represent adaptive mutations of minor effect, or they may be neutral mutations that are either segregating due to drift or have hitchhiked alongside other strongly adaptive mutations. In several cases, MIC analyses suggest a benefit to these mutations arising through increased levels of antibiotic resistance. Genotypes containing a single mutation in Pa14_32420 (encoding a putative oxidoreductase) isolated from an early time point (day 3) showed a 4-fold increase in ciprofloxacin MIC and a SNP in Pa14_46110 (encoding a predicted sodium∶solute symporter), which was the third mutation to arise in the population, had an 8-fold higher MIC than did genotypes carrying only the first two mutations (which occurred in *nfxB* and Pa14_23430). Thus, the evolution of quinolone resistance appears to have involved both highly parallel changes, as well as mutations specific to individual experimental populations.

Previously, Breidenstein *et al.* [48] conducted a screen of transposable-element insertions for novel ciprofloxacin resistance determinants. Interestingly, there is almost no overlap between between the 114 genes identified by Breidenstein *et al.* and the 44 genes bearing SNPs in this study. *nfxB* and *mutS* mutants were isolated in both experiments, but no other gene was found as a potential resistance factor in both studies. In addition, Breidenstein *et al.* identified a number of phage-related or phage-derived genes as resistance modifiers, and we found a non-coding mutation in a different cluster of phage-related genes (at position 1927375 of the Pa14 genome). The difference between these two studies is likely due to the different mechanisms that lead to resistance mutations in the two studies: transposon insertions were used by Breidenstein *et al.* paper, and spontaneous point mutations and indels in the current study. Importantly, the lack of overlap between the two studies is an indication that many genes potentially contribute to fluoroquinolone resistance in *P. aeruginosa*, and suggests that in general multiple approaches should be taken in the identification of genes underlying phenotypes of interest. Experimental evolutionary approaches, such as the one adopted here, differ from traditional mutational studies in that selection acts as an extra sieve that will weed out slow-growing mutants that, while they confer resistance, are out-competed on the way to fixation by other mutations conferring higher fitness (see [17], [18] for discussions of the use of experimental evolution as a tool for mutation discovery). While this effect of natural selection will likely eliminate some mutations of interest (especially for understanding underlying biological pathways), mutations observed under selection may be more clinically relevant due to their relatively high fitness.

### What explains the prevalence of clinical resistance mutations?

Surveys of clinical samples of *Pseudomonas aeruginosa* often uncover a handful of genes with major effects on fluoroquinolone resistance. Most commonly, these genes are *gyrA* and *gyrB*, which encode the subunits of the fluoroquinolone target DNA gyrase, and the efflux pump regulators *nfxB* and *mexR* (e.g., [44], [46], [49], [50]). Given that mutational surveys have revealed many other genes that can confer resistance to fluoroquinolones, why is it that these four genes are repeatedly recovered from clinical samples?

**Figure 5:**
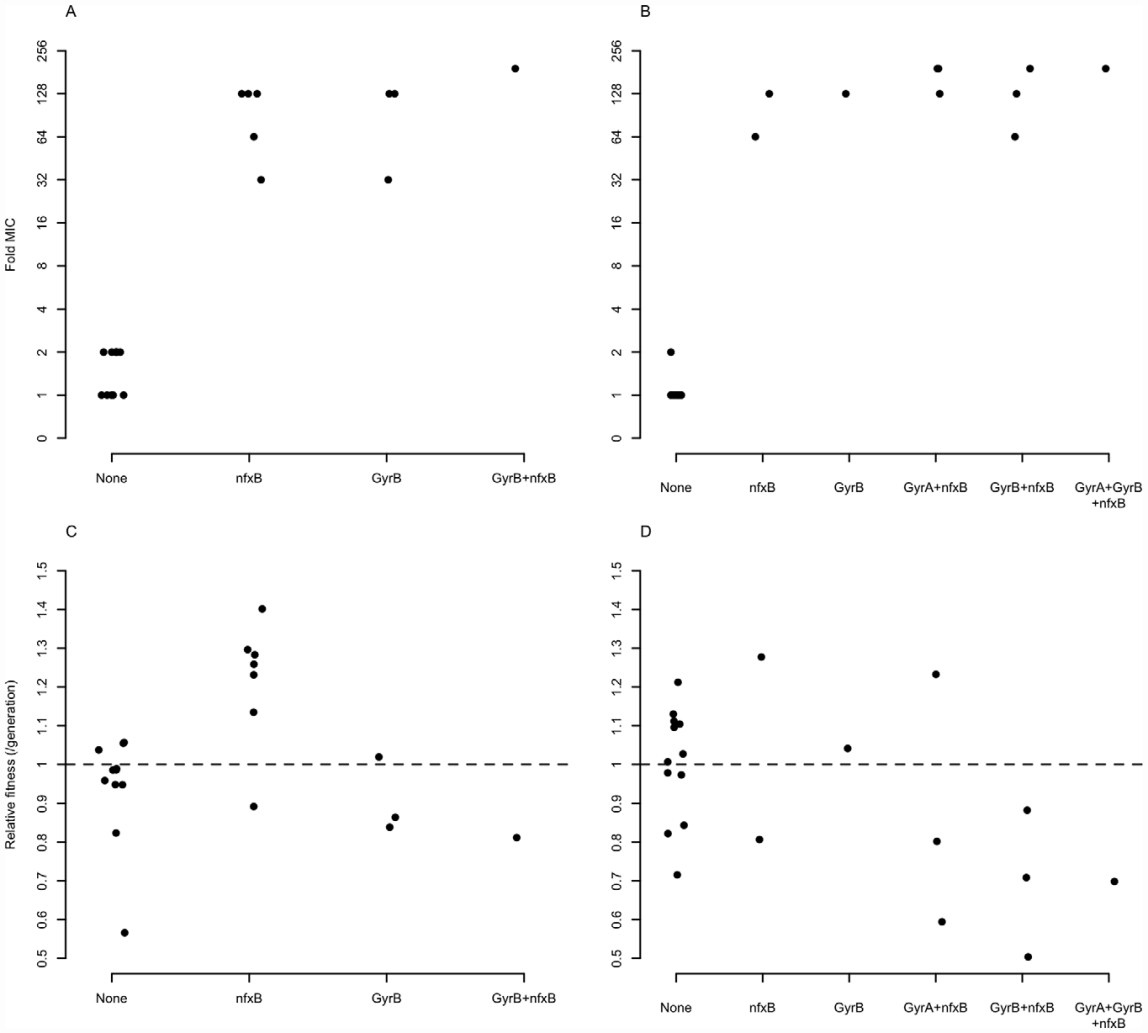
Mutations in known resistance genes are strong predictors of ciprofloxacin resistance, but not fitness. Fold-increase in MIC (A and B), or relative fitness (C and D), for genotypes bearing the given mutations, with genotypes evolved in scfm (A and C) or scfm+mucin (B and D).

One possibility is that these genes enjoy a large fitness advantage in the presence of antibiotic because they confer large increases in MIC. To test this prediction, we asked to what extent the presence or absence of mutations in classical resistance genes is a predictor of the level of ciprofloxacin resistance. As described above, many of the ciprofloxacin-evolved strains in this study bore mutations in one or several of *gyrA*, *gyrB*, and *nfxB*, although no *mexR* mutants were isolated. A linear model including selection medium (scfm+Cip or scfm+mucin+Cip) and presence or absence of mutations in *gyrA*, *gyrB*, and *nfxB* explains ∼87% of variation in MIC between genotypes (Table 2, Figure 5A, 5B). Under this model, mutations in *nfxB*, *gyrA*, and *gyrB* are associated with average MIC increases of 25.3, 3.2, and 10.9-fold, respectively. Thus, a substantial fraction of variation in the level of resistance is attributable to mutations in classical resistance genes. It should be noted that the genotypes indicated in Figure 5 are not exhaustive – for example, a given *nfxB* mutant on Figure 5 will also carry at least one additional mutation. Thus, variation within a genotype class (for example, the *nfxB* mutants) is attributable to these additional mutations.

**Table 2:** Effects of medium and genotype on ciprofloxacin MIC.

Factor	Estimate ( log_2_(MIC) )	Std. Error	t	*P*-value
Intercept	2.1422	0.32	6.63	**8.78×10^−8^**
Medium (mucin)	−0.892	0.41	−2.15	**0.038**
*gyrA*	1.66	0.78	2.13	**0.04**
*gyrB*	3.44	0.49	6.98	**2.98×10^−8^**
*nfxB*	4.66	0.47	9.96	**5.06×10^−12^**

An alternative, and not mutually exclusive, possibility is that these mutations pay little cost of resistance in the absence of antibiotic. Cost-free resistance may arise because the mutations themselves are not costly or because second-site mutations rapidly evolve that compensate for whatever cost they do incur. We tested this prediction by examining the fitness of strains bearing (or not) mutations in classical resistance genes in the absence of ciprofloxacin and found little relationship between genotype and fitness (Table 3, Figure 5C, 5D; see also Figure S6). Notably, only strains carrying *nfxB* mutations from the scfm+Cip environment show an increase in fitness in the absence of antibiotic (Table 3) and none of the *gyrA*, *gyrB*, or *nfxB* mutants from the scfm+mucin+Cip environment were significantly different from the ancestor. This result may be surprising, given that single mutations in *gyrA* and *nfxB* are typically costly [16], [51], [52] but we note that none of the strains examined here carried only a *gyrA* or *nfxB* mutation; all were at least double mutants. This result suggests that fitness in the absence of antibiotic appears to be determined or modulated by mutations in genes other than *nfxB*, *gyrA*, and *gyrB*. Thus cost-free resistance probably arises through second-site mutations that compensate for the costs incurred by these classical resistance genes, consistent with the results of previous studies [13], [53]–[56]. It is notable that these compensatory mutations would have to have arisen very quickly alongside or soon after resistance had evolved for them to be observed in the short time frame of our experiment.

**Table 3:** Effects of genotype on fitness in the absence of antibiotic.

	scfm		scfm+mucin	
Factor	Estimate (*s*)	*P*-value	Estimate (*s*)	*P*-value
Intercept	−0.036	0.41	0.018	0.74
*gyrA*	NA	NA	−0.18	0.15
*gyrB*	−0.14	0.12	−0.076	0.54
*nfxB*	0.22	**0.004**	−0.066	0.64

What sorts of second-site mutations might be involved in compensating for the fitness costs of *nfxB*, *gyrA*, or *gyrB* resistance mutations? Our genome-wide survey of mutations provides some insight. We have found a wide range of mutations amongst the Cip-resistant genotypes sequenced in this study. These include mutations in the gene *nusA* encoding an elongation factor, a putative kinase encoding gene Pa14_28895, and *ate1*, which encodes an arginyl-trNA-protein transferase (see Table S2 for a full list).

While genotype at classical resistance genes predicts MIC (but generally not fitness), we find no evidence that the raw number of mutations present in a lineage predicts either MIC or fitness in the absence of antibiotic (data not shown). These data are consistent with a model in which classical resistance genes make particularly large contributions to MIC that can mask the smaller effects of other resistance mutations, even if these latter mutations occur first or provide additional increases to MIC or fitness.

Taken together, these results suggest that the prevalence of classical fluoroquinolone resistance mutations such as those in *gyrA* and *nfxB* in clinical isolates is due to the combination of high levels of resistance and apparent lack of costs due to second site mutations. These results are of clinical importance because they suggest that attempts to combat resistance in patient populations by stopping the use of the offending antibiotic in the hopes that drug sensitive types will replace resistant ones will often fail (e.g., [57]). Epidemiological evidence on the effectiveness of this strategy at controlling resistance is both limited and mixed [6], [58]: reducing the use of antibiotics often leads to a reduction in the frequency of resistant strains, but it rarely succeeds in eliminating them altogether [4], [5]. Our results suggest that the mechanistic reason for this failure is not that resistance mutations are cost-free but, rather, that their costs are rapidly compensated for by a diverse array of mutations elsewhere in the genome.

### Genetic basis of adaptation to a CF–like culture environment

Our genomic analysis also sheds light on the genetic pathways to adaptation in CF-like conditions. Strains evolved in the most CF-like environment, scfm+mucin, often contained mutations in genes implicated in cyclic-di-GMP signalling. Elevated levels of intracellular cyclic-di-GMP are thought to induce a shift from a motile, planktonic lifestyle to a non-motile biofilm state in a variety of bacteria [27]–[29]. We suspect that increases in diguanylate cyclase activity may be adaptive in the presence of mucin, which encourages biofilm growth. Consistent with this hypothesis, three genes with putative roles in diguanylate cyclase signalling were repeatedly found mutated in the evolved strains. 9 of 24 populations (8 without ciprofloxacin, 1 with ciprofloxacin) contained isolates bearing mutations in the *morA* gene (Figure 4). *morA* encodes a predicted membrane-localized diguanylate cyclase, and serves as a negative regulator of flagellum formation [59]. In *P. aeruginosa*, expression of *morA* is required for the switch from wild-type colony morphology to the small-colony variant morphology [27], which is associated with biofilm formation in CF infections [25]. 7 distinct *morA* mutations – all missense point mutations - were identified in our evolved strains (Figure 4). Two scfm+mucin-evolved strains bore mutations in *wspF*, which encodes a regulator of the diguanylate cyclase WspR, with *wspF* loss-of-function mutants showing increased biofilm formation [28] and wrinkly colony morphologies in *Pseudomonas fluorescens* [60]. One of the *wspF* alleles recovered in this study is likely a loss-of-function mutation, since it encodes an early frame-shift. The second allele is a single in-frame codon deletion whose effects we cannot predict. Finally, the gene Pa14_56280, encoding another predicted diguanylate cyclase, was found to be mutated in two further scfm+mucin adapted strains.

**Figure 6:**
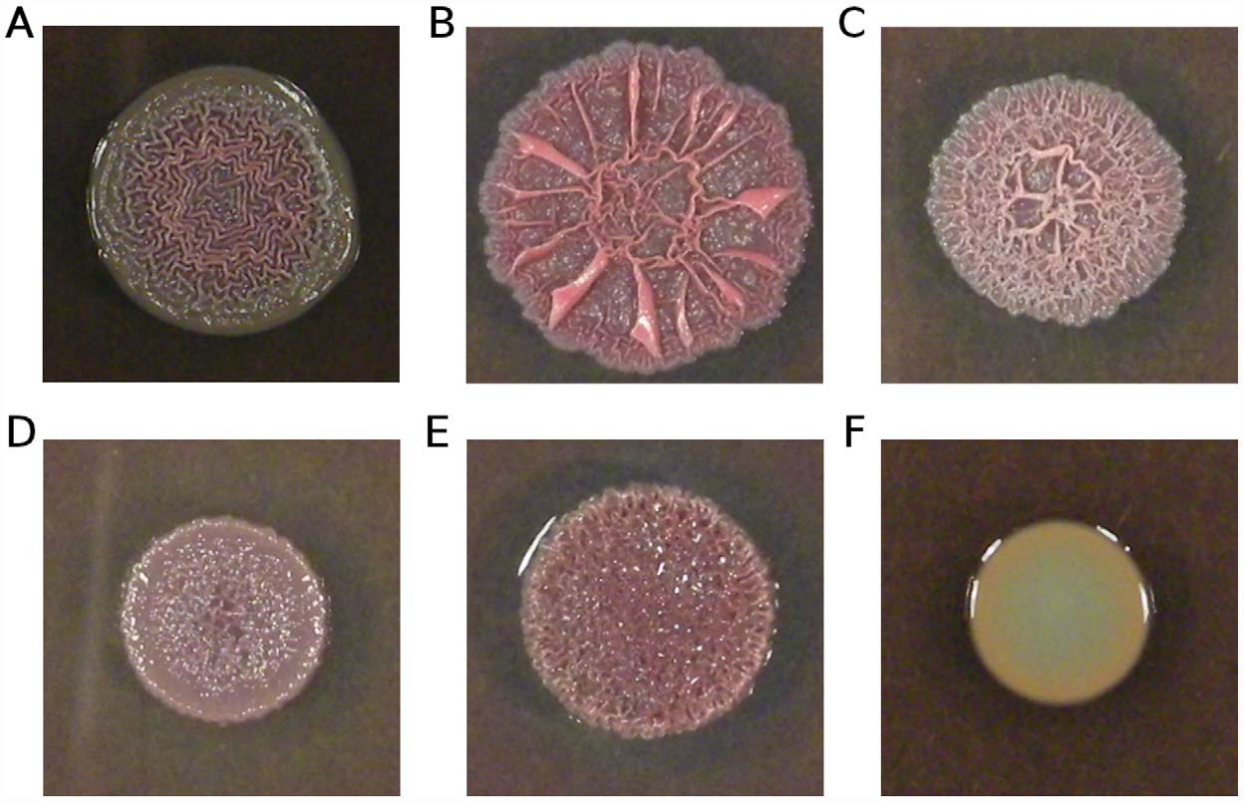
Variation in colony morphology. Isolates were grown on 1% tryptone plates containing Congo red and Coomassie blue. (A) Ancestral Pa14; (B) strain smA1 carrying a single mutation in *morA* (H975Y); (C) strain smA2 carrying a different mutation in *morA* (L1155Q); (D) strain smD6 bearing three mutations, including one in *wspF* (an out-of-frame deletion); (E) strain smC3 bearing a single mutation in Pa14_56280 (M204I); (F) strain scfmB5 carrying a single mutation in *nfxB* (G180S).

In light of the role of cyclic-di-GMP signalling in biofilm formation [27]–[29], we predicted that our putative cyclic-di-GMP signalling mutants should show increased aggregation and biofilm formation. To test this prediction, we examined colony morphology on Coomassie blue/Congo red agar plates, which is a sensitive indicator of aggregation (e.g., [61]–[63]). Isolates bearing mutations in *morA*, *wspF*, or Pa14_56280 showed wrinkly, red morphologies in comparison to the ancestral Pa14 strain (Figure 6A–6E), consistent with increased aggregation and biofilm formation. Genotypes bearing mutations in different genes, and even different mutations in the same gene (e.g. for *morA*, compare Figure 6B and 6C), showed different colony morphologies, suggestive of different effects on the level, timing, and/or localization of aggregation signals, presumably cyclic-di-GMP.

The frequency with which cyclic-di-GMP signalling genes are mutated in our mucin evolved strains – with apparent consequences for aggregation and biofilm formation – strongly suggests a shared mode of adaptation towards a novel *in vitro* environment. This finding parallels data from clinical isolates of *P. aeruginosa*: Long-term adaptation of *P. aeruginosa* to the CF lung is characterized in part by extensive biofilm formation (e.g., [26], [64]) and the switch to a largely non-motile lifestyle is likely mediated by cyclic-di-GMP signalling (e.g., [25], [65]). Notably, *wspF* mutations have previously been documented in CF isolates (e.g., [10]); the current data suggest several other possible mediators of biofilm formation in clinical isolates.

Unexpectedly, all strains bearing mutations in the quinolone-resistance gene *nfxB* showed smooth colony morphologies (Figure 6F), a phenotype typically associated with impaired biofilm production (e.g., [61]–[63]). This observation suggests an effect of *nfxB* on biofilm formation and/or extracellular matrix production, which to our knowledge has not been previously reported.

### Parallel evolution

The extent of parallel evolution during adaptation is of interest for a variety of reasons; evolution is in principle predictable (or not) to the extent that independent populations adapt to similar environments via the same (or different) mutations. The observation of substantial parallel evolution is also used as an indicator of strong positive selection. Previous experimental evolution studies have documented varying degrees of parallel evolution at both the phenotypic and genotypic levels [66]–[73]. We have already noted parallel evolution in response to ciprofloxacin and to mucin in our study, with multiple lineages bearing mutations in the quinolone resistance genes *gyrA*, *gyrB*, *nfxB*, *orfN*, and in the apparently mucin-adaptive genes *morA*, *Pa14_56280*, and *wspF*. These observations provide strong evidence that these mutations are beneficial.

How prevalent is parallel evolution in our study? To answer this we used the Jaccard index (*J*) to quantify the extent of within- and between-environment genic parallel evolution. For a given pair of evolved genotypes, *J* ranges from 0 to 1, with 0 indicating no parallel evolution and 1 indicating identity (see Materials and Methods for further details). We calculated the average Jaccard index 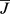 for within- and between-environment comparisons, excluding genotypes with no SNPs, as well as *mutS* mutator strains (Figure S7). Within environments, 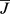 was highest for the scfm+mucin genotypes, due to the high frequency of *morA* mutations in this environment. 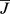 was intermediate for the scfm+Cip and scfm+mucin+Cip genotypes, reflecting parallel evolution at a handful of genes combined with a number of lineage-specific mutations. Between-environments, 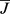 was 0, except for between the two ciprofloxacin treatments, indicative of some shared mechanisms of resistance. We rarely saw the exact same mutation evolving in parallel selection lines, suggesting that the bulk of parallel evolution in our experiment is through *de novo* mutations rather than the selection of rare, pre-existing variants. For the few cases where the same mutation was observed in multiple lineages, however, we note that the current study design cannot formally distinguish between these two alternatives since our experimental populations were started from a common founding culture.

We suspect that several different factors contribute to differences in the propensity for parallel evolution at different genes. Chevin *et al.* [74], analyzing an explicitly genomic model of trait evolution, show that the probability of parallel evolution at a given locus can depend on the locus specific mutation rate, the probability of a mutation being beneficial, and the probability of a mutation going to fixation. For some loci, e.g. *nfxB*, loss-of-function mutations are likely to be beneficial, and so the probability of a mutation being beneficial will be quite high (see [73] for a similar example). For other loci, such as *gyrA*, the probability of fixation for beneficial mutations may be high due to their large effects on MIC. Finally, in the case of *orfN*, where a slippage mutational mechanism is implicated by the observation of single base deletions in repeat regions, both the mutation rate and the probability of a mutation being beneficial are likely to be elevated. Thus, different genes may undergo parallel evolution for rather different reasons.

### Summary and conclusions

We have studied the genomic basis of adaptation to CF-like culture conditions and to ciprofloxacin in experimentally evolved isolates of the opportunistic pathogen *P. aeruginosa*. Adaptation did occur to the most CF-like conditions and to the presence of ciprofloxacin, although our evolving populations are likely highly polymorphic. We observed parallel evolution at a handful of antibiotic resistance genes (*gyrA*, *gyrB*, *nfxB*, and *orfN*), as well as at putative cyclic-di-GMP signalling genes in the mucin environment. While the level of antibiotic resistance was determined largely by known resistance genes, fitness in the absence of antibiotic was not, such that there was no overall relationship between resistance and its associated costs.

These findings have several implications for understanding antibiotic resistance and pathogen evolution. First, we have identified a suite of novel ciprofloxacin resistance mutations. Our evolved antibiotic resistant isolates harbour mutations in 12 genes not previously implicated in fluoroquinolone resistance, and initial assays are consistent with effects on ciprofloxacin MIC for 3 of these genes (*orfN*, Pa14_46110, and Pa14_32420). Thus, experimental evolution, coupled with WGS, represents a powerful approach to identifying novel genes of interest.

Second, we find that the costs of resistance are not systematically determined by the same mutations that account for most of the variation in level of resistance (i.e., mutations in *gyrA*, *gyrB*, and *nfxB*). This finding suggests that whatever costs are associated with single resistance mutations are easily remediated by mutations at other loci. Moreover, these results suggest that the prevalence of these resistance mutations in clinical isolates are likely the result both of the high levels of resistance they confer and the rapid compensation of costs by second-site mutations.

Third, the finding of multiple cyclic-di-GMP mutations in the mucin environment underscores the importance of GMP-mediated biofilm formation in viscous environments, such as the CF lung.

Finally, our findings suggest that pathogen evolution has a partially repeatable genomic basis, insofar as some genes are repeatedly mutated in multiple replicate populations, while others are not. This observation has important implications for understanding pathogen evolution. Those genes that show highly parallel evolution may be particularly important in their influence on key adaptive traits governing infection or resistance to antibiotics. However, genes that are mutated only rarely are not necessarily unimportant: they often appear to have important phenotypic consequences, such as compensating for costs of resistance, and so cannot be ignored. In designing novel medical interventions, therefore, our results suggest that we would do well to focus attention first on these common targets of adaptation to the lung environment, while not losing sight of the potential importance of rare and sometimes idiosyncratic mutations that nevertheless play a major role in determining the overall fitness of the pathogen.

## Materials and Methods

### Experimental evolution

A single colony of *P. aeruginosa* strain Pa14 was grown overnight in minimal medium (NH_4_Cl 1 g/L, KH_2_PO_4_ 3 g/L, NaCl 0.5 g/L, Na_2_HPO_4_ 6.8 g/L; supplemented with CaCl_2_ 15 mg/L, MgSO_4_ 0.5 g/L; 0.8% dextrose as a carbon source). Forty-eight populations were founded from this progenitor by adding 25 µL overnight culture to 1.5 mL of fresh medium (media described below). An aliquot of progenitor was frozen at −80°C in glycerol. Populations were grown on an orbital shaker (150 rpm) at 37°C for 24 hours in 24-well plates. After 24 hours, each population was serially propagated by transferring 25 µL of overnight culture to 1.5 mL of fresh medium. Overnight cultures were frozen at −80°C in glycerol. Seven such transfers were conducted in total, such that approximately 50 generations of evolution occurred (∼5.9/day for 8 days).

Four selection environments were used, consisting of two different media with or without antibiotic. The media were chosen so as to examine the effects of CF sputum nutrition and viscosity on the evolution of antibiotic resistance in *P. aeruginosa*. Synthetic CF sputum (scfm) was prepared as described by [30]). In order to manipulate viscosity, we added 10 g/L porcine mucin (Sigma) to synthetic CF sputum (scfm+mucin)[32], [33]. For antibiotic treated populations, we used 1 µg/mL ciprofloxacin to mimic the concentration typically found in the sputum of CF patients [31].

### Phenotypic analyses

For each evolved population, or for pure genotypes isolated from each population, level of resistance was assayed as the minimal inhibitory concentration (MIC) of ciprofloxacin. Overnight cultures were grown in Mueller-Hinton broth (MHB; Sigma), of which 5 µL was inoculated into 195 µL of fresh MHB with varying concentrations of ciprofloxacin in 96 well plates. MIC of the ancestor, i.e., the concentration at which growth was inhibited by 90%, was 0.05 µg/mL. For each evolved strain, we assayed growth at 0x, 0.5x, 1x, 2x, 4x, 8x, 16x, 32x, 64x, 128x, 192x, and 256x the ancestral MIC.

Fitness of each evolved population or genotype was assayed using a competitive fitness assay against a *lacZ* marked ancestral strain. Independent assays verified that the *lacZ*-marked strain did not bear a fitness cost in competitions with unmarked Pa14. Both competitors were grown for 24 hours in the competition medium. At time 0, 12.5 µL of marked ancestor and 12.5 µL of evolved strain were inoculated into 1.5 mL of fresh medium in a 24-well plate, and an aliquot was frozen at −80°C in glycerol. Following 24 hours of growth at 37°C at 150 rpm, a final aliquot was frozen at −80°C in glycerol. Serial dilutions of initial and final aliquots were grown on solid minimal media+X-gal, allowing us to determine the numbers of blue (ancestral) and white (evolved) individuals at the beginning and end of the competition. The selection coefficient *s* was calculated as:
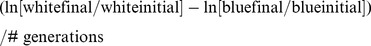
Relative fitness *w* was then calculcated as 1+*s*, where the units for both *w* and *s* are in per generation.

Colony morphology was assayed according to [61]. Briefly, 10 µL of culture grown overnight in LB were spotted in triplicate onto tryptone plates (10 g/L) supplemented with 20 µg/ml Coomassie blue and 40 µg/ml Congo red. Plates were grown for 4 days at room temperature, after which digital photos were taken.

### Whole-genome sequencing and analysis

For whole-genome sequencing, a single colony was picked from each evolved population, as well as for the ancestral Pa14 genotype. For each genotype, genomic DNA was extracted from an overnight culture using the Promega Wizard Genomic DNA Purification kit. 75-bp paired-end Illumina sequencing was performed by the Michael Smith Genome Sciences Centre, using DNA barcodes to sequence 10–12 isolates per lane. Mean coverage across all 49 genotypes was 55.5-fold at a quality score of 20 (range: 31.8–85.4).

We performed a pair-end mapping of reads on the Pa14 reference genome number NC_008463.1 using *novoalign* (http://novocraft.com/main/index.php). We used samtools [75] to call snps/indels, and filtered the resulting calls using the provided *samtools.pl* script, changing the window size for snps around indels at 5 base pairs, removing the limit on number of reads spanning a snp/indel position, and leaving the remaining parameters at their default values. We further filtered calls with quality scores below 60 for indels, and 20 for snps. To annotate the remaining snps/indels with respect to the reference genome, we used snpEff (http://snpeff.sourceforge.net/). We found results to be robust to performing a pre-mapping clipping of reads based on quality across cycles using FastQC (http://www.bioinformatics.bbsrc.ac.uk/projects/fastqc/), and to performing local multiple sequence re-alignment around indels using the Genome Analysis ToolKit [76]. We also used the BRESEQ [38] pipeline as a further validation, and for its insertion/deletions detection capabilities.

Following removal of common assembly errors using custom perl scripts, a subset of SNPs was verified by Sanger sequencing of polymerase chain reaction (PCR) amplicons. For each of 31 mutations (out of 98 mutations identified in the 48 evolved strains), we amplified a 500–700 bp PCR product containing the putative SNP, and directly sequenced the PCR products (Genome Quebec, Montreal). All 31 mutations that we interrogated were successfully verified.

We used a randomization approach to determine the probability of observing by chance the distribution of non-synonymous, synonymous, and intergenic point mutations. This analysis was performed separately for putative mutator strains (two *mutS* mutants) and for putative non-mutator strains (the remaining 46 strains). 10 000 sets of point mutations were generated at random from the Pa14 genome sequence, maintaining the observed numbers of transitions and transversions (mutators: 30 transitions and 11 transversions; non-muators: 26 transitions and 0 transversions), and SNP effects were predicted using snpEff. Mean numbers of non-synonymous, synonymous, and intergenic mutations, as well as the 2.5% and 97.5% quantiles of the random distribution, were calculated in R [77].

### Parallel evolution

The extent of parallel evolution was quantified using the Jaccard Index *J*. Given two sets *G*_1_ and *G*_2_ that list mutation-bearing genes found in genotypes 1 and 2, respectively,
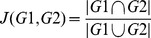
That is, *J* is the number of genes mutated in both strains divided by the total number of genes mutated in genotype 1 or in genotype 2. *J* ranges from 0 to 1, with 1 indicating identical genotypes and 0 indicating no shared mutations.

*J* was calculated for all possible pairs of different genotypes amongst the 46 non-mutator strains. The average Jaccard Index 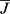 was calculated within a treatment group as the mean *J* for all pairs of strains, where both strains were evolved under the same treatment. Similarly, 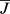 was calculated between treatments A and B as the mean *J* for all pairs of strains, where one strain was evolved under treatment A and the second strain was evolved under treatment B.

## Supporting Information

Figure S1 No relationship between pure genotype fitness and population fitness. Each panel gives a dashed 1∶1 line, and a solid regression line.
(JPG)

Figure S2 Pure genotype MIC is predicted by population MIC in scfm+mucin, but not scfm. Each panel gives a dashed 1∶1 line, and a solid regression line.
(JPG)

Figure S3 Genomic locations of mutations identified by whole-genome sequencing. Mutations found only in putative mutator strains (*mutS* mutants) are not shown.
(PNG)

Figure S4 Expected and observed distributions of non-synonymous, synonymous, and intergenic mutations. Bar plots show the expected mean and 2.5% and 97.5% quantiles (error bars) for randomly generated mutations, with observed data represented by solid black points. (A) Non-mutator strains only. (B) Mutator strains only.
(JPG)

Figure S5 Site-frequency spectrum of mutations identified in non-mutator evolved strains.
(JPG)

Figure S6 Higher resistance does not correlate with greater costs of adaptation. No correlation between MIC and fitness in the absence of antibiotic in scfm or in scfm+mucin. Single gentoype data are given in the top two panels, and population data are given in the bottom two panels.
(JPG)

Figure S7 Parallel evolution within and between treatements. Parallel evolution was quantified as the average Jaccard index. Values are given within treatments (circles) or between treatments (lines).
(JPG)

Table S1 Mutations detected by Illumina sequencing of evolved genotypes. Strain names give the selection medium as scfm or sm (scfm+mucin). Strain names ending in 1–3 were evolved in the absence of Cip, while those ending in 4–6 were evolved in the presence of Cip. Putative mutator strains are highlighted in red.
(XLS)

Table S2 Mutations with possible compensatory effects on classical resistance genes. Mutations found to co-occur with known resistance mutations, that may reduce or eliminate costs associated with Cip resistance.
(XLS)
