# Supplementary figures and images for: Genomics of Adaptation during Experimental Evolution of the Opportunistic Pathogen Pseudomonas aeruginosa
Source: PLoS Genet. 2012 Sep 13;8(9):e1002928. doi: 10.1371/journal.pgen.1002928 (PMC3441735; doi:10.1371/journal.pgen.1002928)

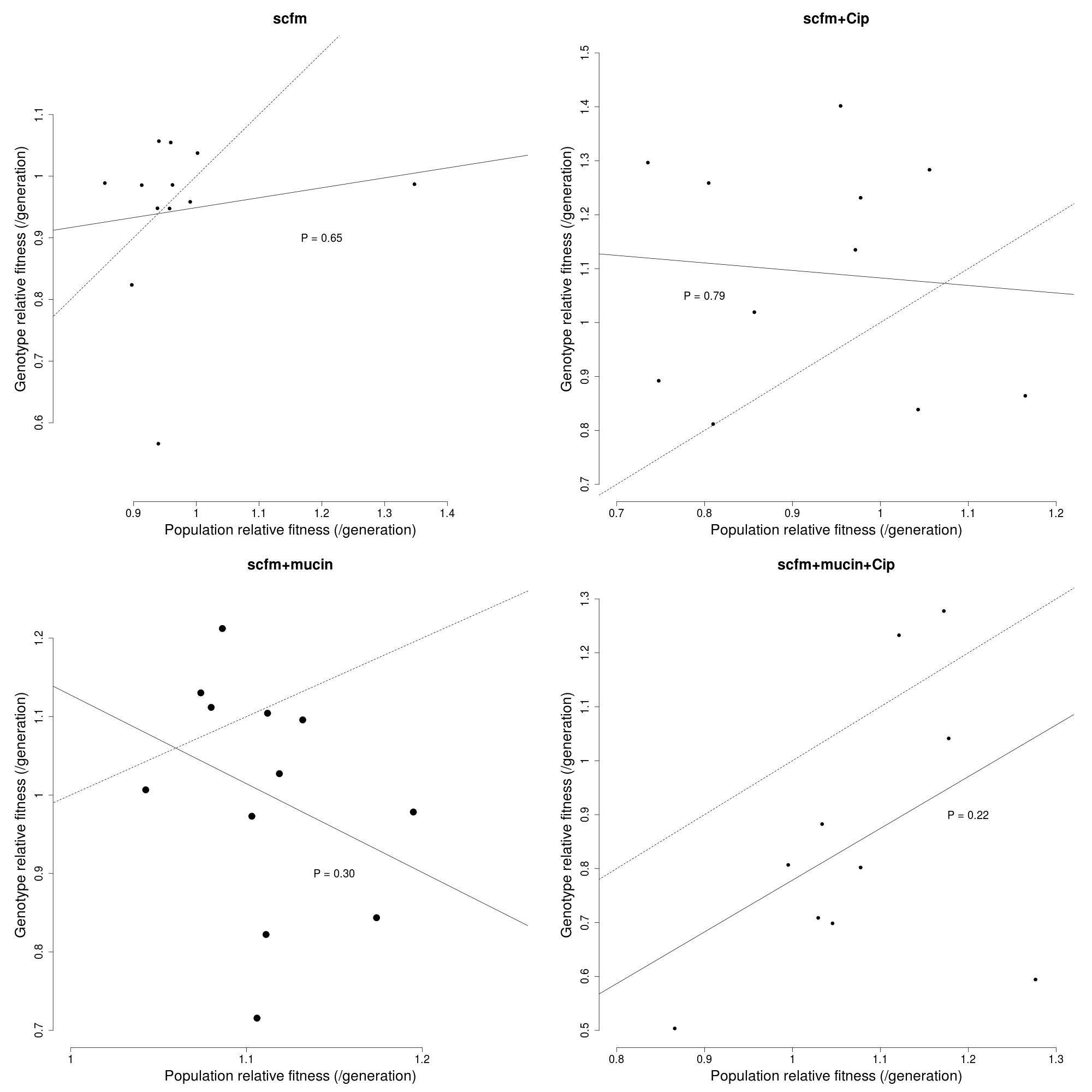

Supplement: Figure S1 — No relationship between pure genotype fitness and population fitness. Each panel gives a dashed 1∶1 line, and a solid regression line. (JPG) [file pgen.1002928.s001.jpg]

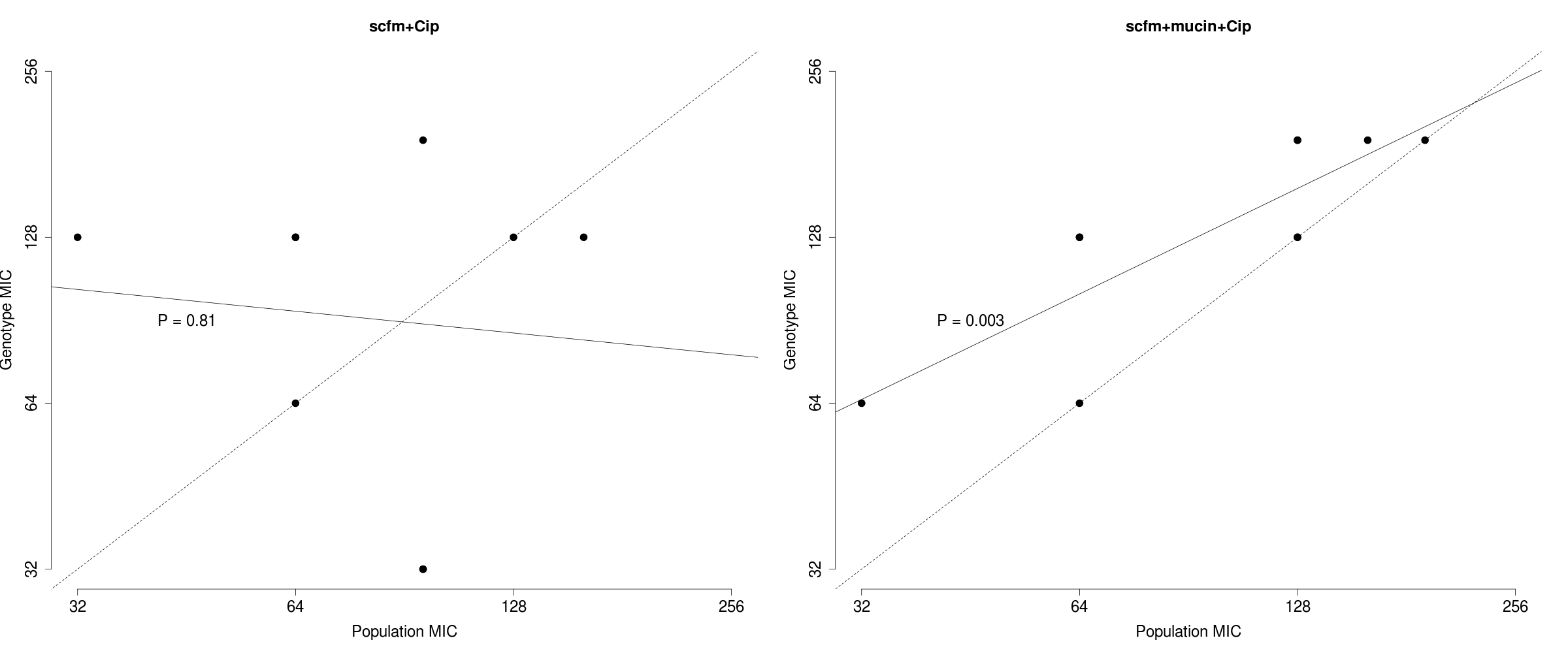

Supplement: Figure S2 — Pure genotype MIC is predicted by population MIC in scfm+mucin, but not scfm. Each panel gives a dashed 1∶1 line, and a solid regression line. (JPG) [file pgen.1002928.s002.jpg]

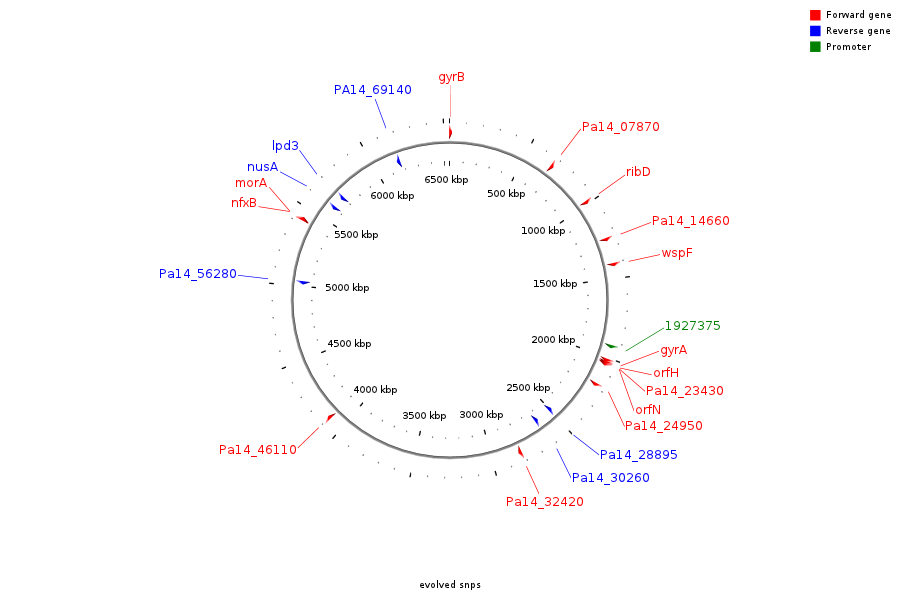

Supplement: Figure S3 — Genomic locations of mutations identified by whole-genome sequencing. Mutations found only in putative mutator strains (mutS mutants) are not shown. (PNG) [file pgen.1002928.s003.png]

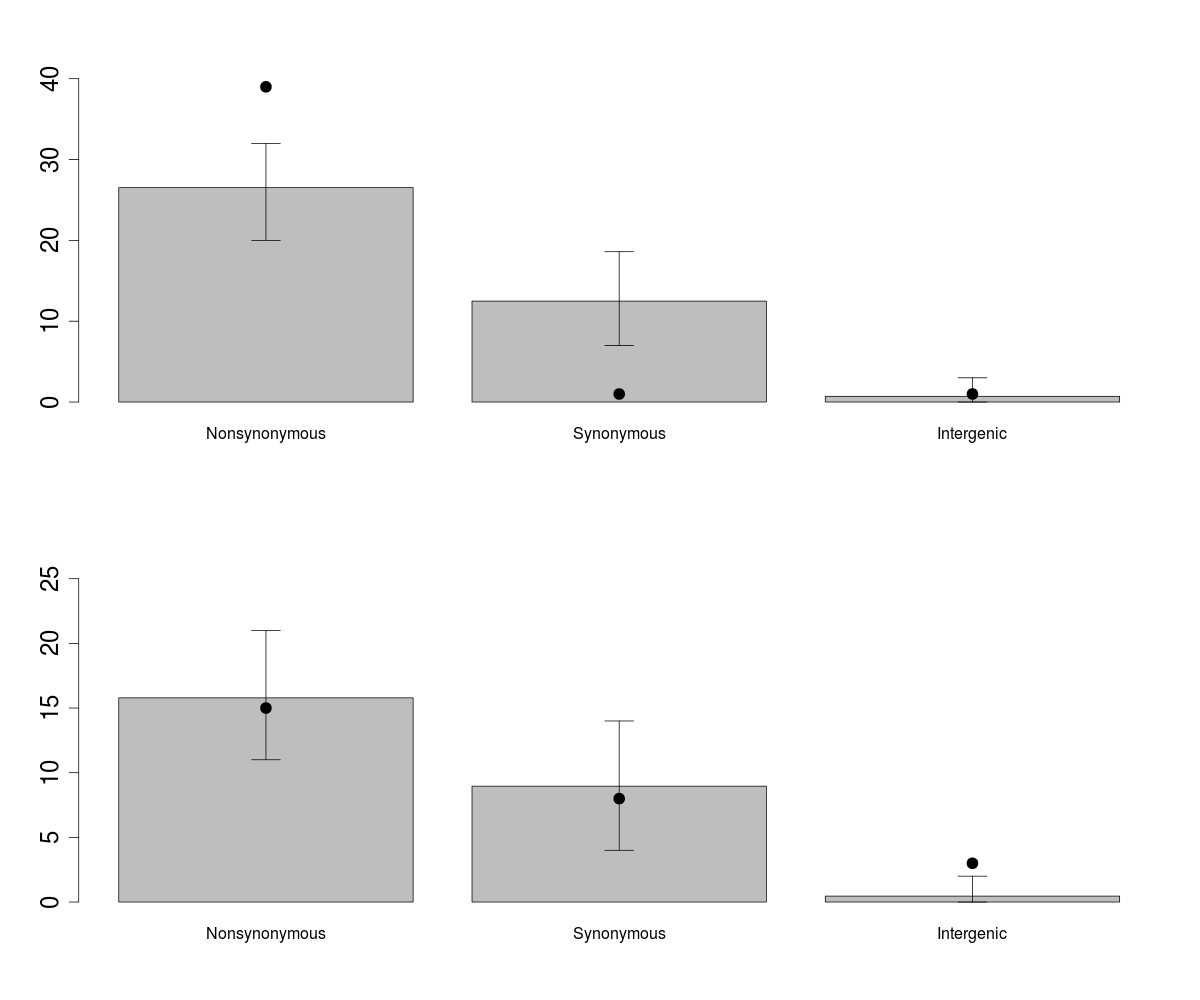

Supplement: Figure S4 — Expected and observed distributions of non-synonymous, synonymous, and intergenic mutations. Bar plots show the expected mean and 2.5% and 97.5% quantiles (error bars) for randomly generated mutations, with observed data represented by solid black points. (A) Non-mutator strains only. (B) Mutator strains only. (JPG) [file pgen.1002928.s004.jpg]

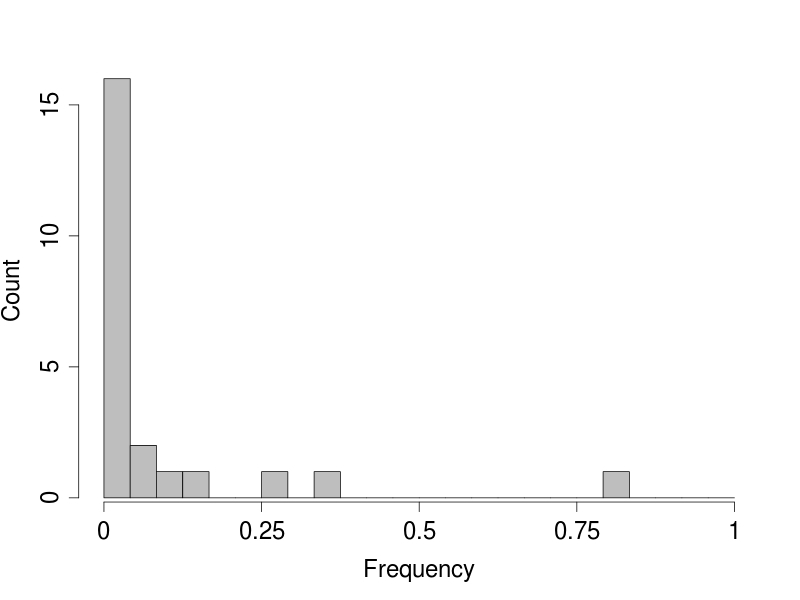

Supplement: Figure S5 — Site-frequency spectrum of mutations identified in non-mutator evolved strains. (JPG) [file pgen.1002928.s005.jpg]

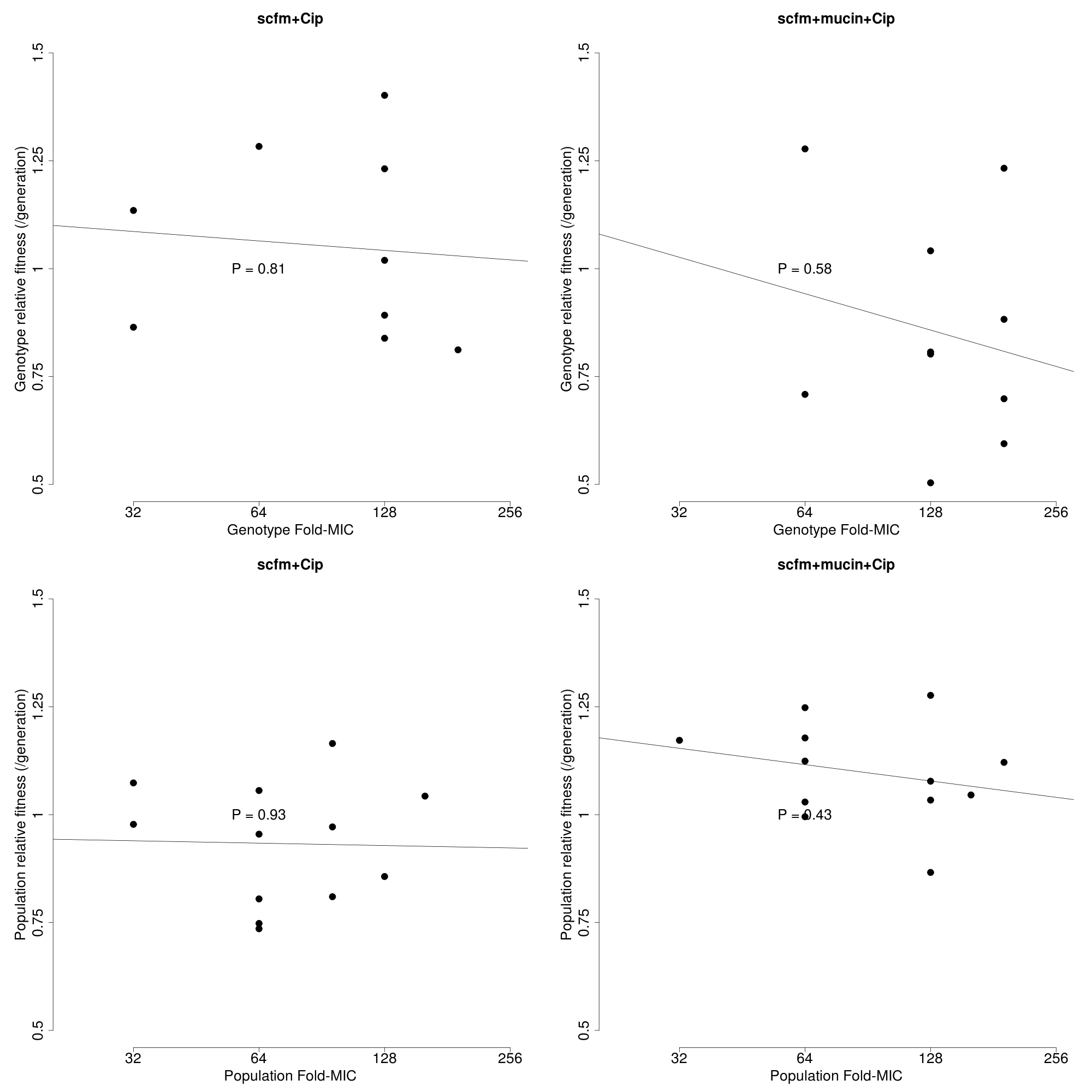

Supplement: Figure S6 — Higher resistance does not correlate with greater costs of adaptation. No correlation between MIC and fitness in the absence of antibiotic in scfm or in scfm+mucin. Single gentoype data are given in the top two panels, and population data are given in the bottom two panels. (JPG) [file pgen.1002928.s006.jpg]

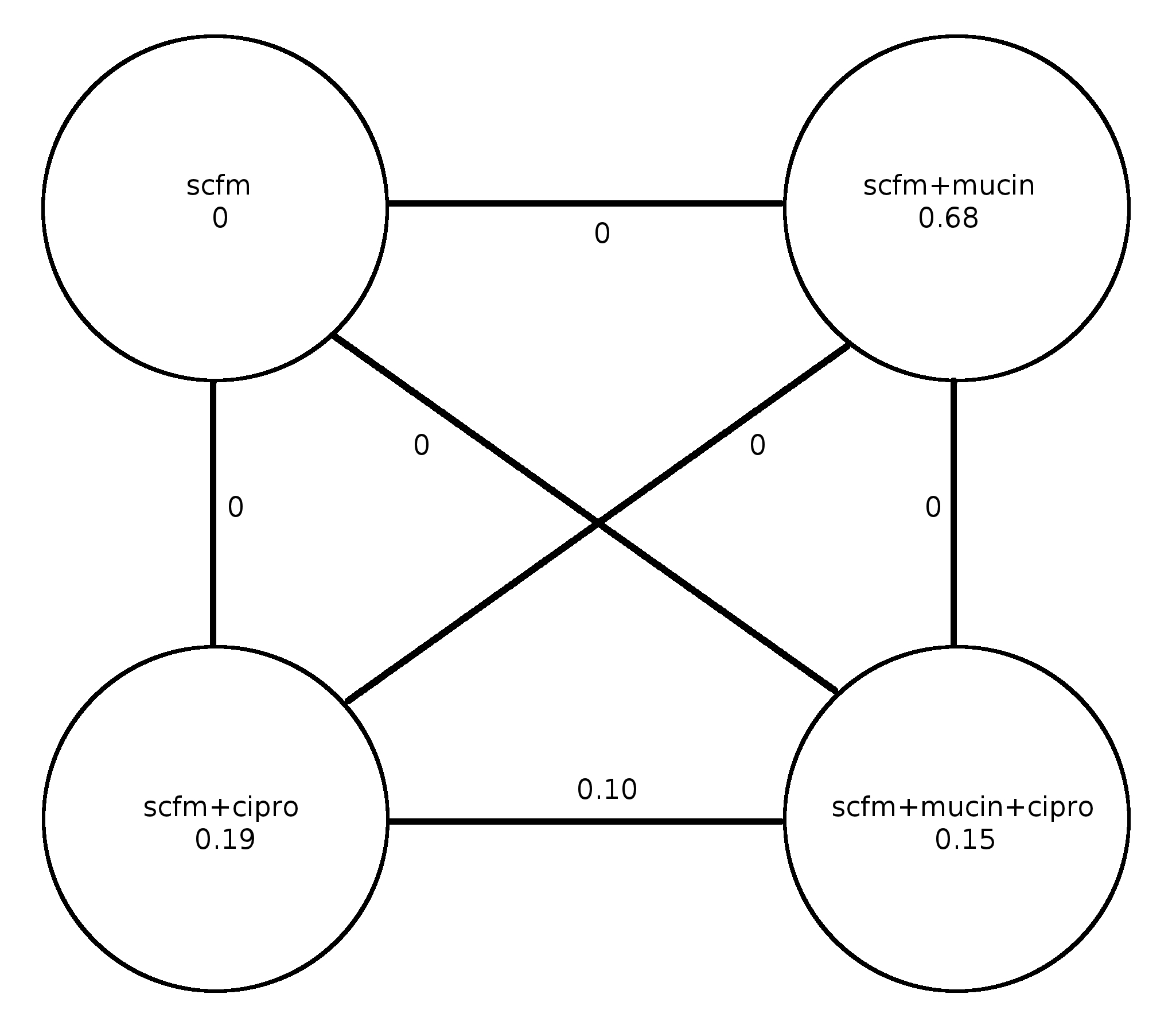

Supplement: Figure S7 — Parallel evolution within and between treatements. Parallel evolution was quantified as the average Jaccard index. Values are given within treatments (circles) or between treatments (lines). (JPG) [file pgen.1002928.s007.jpg]
